# An implementation science protocol of the Women’s Health CoOp in healthcare settings in Cape Town, South Africa: A stepped-wedge design

**DOI:** 10.1186/s12905-017-0433-8

**Published:** 2017-09-18

**Authors:** Wendee M. Wechsberg, Jacqueline W. Ndirangu, Ilene S. Speizer, William A. Zule, Winnifred Gumula, Courtney Peasant, Felicia A. Browne, Laura Dunlap

**Affiliations:** 1 RTI International, 3040 Cornwallis Road, Research Triangle Park, NC 27709 USA; 2 Gillings School of Global Public Health, University of North Carolina at Chapel Hill, CB #7445, Chapel Hill, NC 27599 USA; 3 Department of Psychology, North Carolina State University, 2310 Katharine Stinson Drive Raleigh, Raleigh, NC 27607 USA; 4 Psychiatry and Behavioral Sciences, Duke University School of Medicine, 40 Duke Medicine Cir, Durham, NC 27710 USA; 5 RTI International, 701 13th St. NW #750, Washington, DC 20005 USA; 6 Kheth’Impilo, 11th floor Metlife Centre, 7 Walter Sisulu Avenue, Cape Town, 8001 South Africa

**Keywords:** HIV prevention, ART adherence, Women, Substance use, Healthcare settings

## Abstract

**Background:**

HIV persists as a public health emergency in South Africa, especially among women of childbearing age. In response to the HIV epidemic, the Joint United Nations Programme on HIV/AIDS has put forth the 90–90-90 global goals to achieve an AIDS-free generation by 2020. This goal aspires to have 90% of people living with HIV diagnosed; 90% of those who test positive on sustained antiretroviral therapy (ART); and 90% of those on ART be virally suppressed. Ensuring access to ART is an important first step in reducing HIV incidence, especially among vulnerable populations such as women who use substances and bear the burden of HIV in South Africa. Additionally, alcohol and other drug (AOD) use and exposure to gender-based violence are associated with increased risk of HIV infection and reduced adherence to ART. However, no research has estimated ART adherence rates for women who use substances in South Africa since the government approved the provision of ART to all people living with HIV.

**Methods:**

The Women’s Health CoOp (WHC) is an evidence-based, woman-focused, behavioral intervention that addresses the intersecting risks of AODs, sex behaviors, and violence and victimization, with the primary goal of increasing skills and knowledge to reduce substance abuse and HIV risks and to improve ART adherence. The WHC has been packaged for further dissemination. This article describes the study protocol used to assess the feasibility and acceptability of implementing the WHC intervention into standard of care in Cape Town health clinics and substance abuse rehabilitation centers to reduce HIV risk behavior and increase ART adherence among women who use substances and are living with HIV.

**Discussion:**

Because few of the interventions that demonstrate efficacy for HIV prevention and ART adherence in randomized trials are sustainable, studies to adapt and test intervention variations are needed to determine the best strategies for implementing them in real-world, high-risk settings. However, implementation in real-world settings presents challenges. Consequently, intervention developers should consider the strengths and limitations of their anticipated implementation setting by engaging with key stakeholders before, during, and after the adaptation and implementation process when developing and attempting to scale-up interventions.

**Trial registration:**

NCT 02733003 and date approved 1/21/2016.

## Background

Mounting evidence indicates that large-scale provision of antiretroviral therapy (ART) is key to arresting the HIV epidemic [1]. Two key studies have demonstrated a decreased incidence of HIV among seronegative partners when their HIV-positive partners initiate ART as soon as they receive their diagnosis [2], and lower HIV prevalence among persons living in areas where there is greater ART use among people who are HIV-positive [1]. In light of the rigorous trials highlighting the overwhelming need to extend distribution of ART, in 2015 the World Health Organization revised its HIV treatment guidelines, recommending that all people living with HIV receive ART regardless of CD4 count [3]. Also, the Joint United Nations Programme on HIV/AIDS (UNAIDS) has put forth the 90–90-90 global goals to achieve an AIDS-free generation by 2020 [4]. This goal states that 90% of people living with HIV are diagnosed, 90% of those who test positive for HIV are on sustained ART, and 90% of those on ART will be virally suppressed [4].

Ensuring access to ART is an important first step in reducing HIV incidence. However, numerous barriers exist between access to ART and actual use and adherence. These barriers often relate to behavioral factors that increase a person’s risk of HIV acquisition or reduce their agency to access or adhere to their medications. In particular, previous research demonstrates that transactional sex, alcohol and other drug (AOD) use, and exposure to gender-based violence (GBV) are associated with increased risk of HIV infection and reduced ART adherence [5–12]. To curb the incidence of HIV and ensure that people living with HIV are able to live longer and healthier lives, it is important to implement evidence-based interventions (EBIs) that address gender context and reduce risk behaviors to help improve ART uptake and adherence. Implementation science provides a framework for determining the best strategies for integrating EBIs into standard healthcare practice and assessing their feasibility, acceptability, and effectiveness as they are rolled out in real-world settings [13].

In this article, we describe the protocol for a study funded by the U.S. National Institute on Alcohol Abuse and Alcoholism to assess the feasibility and acceptability of implementing an evidence-based, gender-focused intervention—the Women’s Health CoOp (WHC) program—in government health clinics that provide HIV testing and counseling (HTC), ART, tuberculosis (TB) treatment, and antenatal care; and in substance use rehabilitation (rehab) centers in South Africa. The project also assesses the effectiveness of the WHC among the women participants and the sustainability of the program in these settings over time.

### A focus on women and the 90–90-90 goal in South Africa

South Africa is the epicenter of the HIV epidemic; evidenced by the fact that 1% of the world’s population lives in South Africa, but about 17% of all persons living with HIV (5.6 million people) reside there [14]. Extremely vulnerable, impoverished women living in South African disadvantaged communities face myriad risks for HIV. For example, historically South Africa has had a patriarchal culture that fueled women’s social, legal, and economic disempowerment [15]; which, in turn, increases their risk of HIV infection. Research from over a decade in Pretoria has demonstrated that HIV prevalence remained stable among women who engage in sex work at about 68%, but increased among women who do not engage in sex work who use substances, including alcohol, from 34% in 2004–2007 to 47% in 2012–2014 [16]. Furthermore, recent research with women who engage in sex work and women who do not engage in sex work in Pretoria indicated that only 16% of women who were HIV-positive were on ART [17].

Achieving the 90–90-90 goal in South Africa will require reaching vulnerable populations, such as women who engage in sex work and/or substance use, and testing them for HIV, linking them to ART, and retaining them in care. Gender-focused, behavioral interventions that address multiple risk-taking behaviors can facilitate ART initiation and retention. However, contextual and structural factors—such as lack of transportation, food insecurity, myths and beliefs regarding HIV, and navigating the public health system—create barriers that interfere with ART initiation and adherence [18]. Additionally, many vulnerable South African women experience GBV or AOD use (or a combination of these factors), which can contribute to HIV risk [9–12, 19]. Furthermore, multiple barriers exist regarding access to and the use of substance use rehab in South Africa, which is further complicated by the scarcity of affordable programs and a lack of knowledge regarding how to access the limited programs that are available [20].

### Addressing the nexus of substance use, gender-based violence, and HIV risk

Since 2001, our research team has developed and adapted the WHC, an evidence-based, woman-focused, behavioral HIV prevention intervention that reaches high-risk, hard-to-reach women in various contexts. The WHC addresses the intersecting risks of substance use, sex behaviors, and violence and victimization, with the primary goal of increasing skills and knowledge to reduce substance abuse and HIV risks. The original Women’s CoOp intervention was conducted in the United States and was named a Centers for Disease Control and Prevention “best-evidence” behavioral intervention for HIV prevention [21]. Since its inception, it has been adapted from previous iterations to new populations and settings in North Carolina, Rhode Island, Massachusetts, Russia, the Republic of Georgia, and several regions in South Africa [15, 22–27]. To promote the use of EBIs in Africa, the U.S. Agency for International Development (USAID) included the first adaptation of the WHC intervention [22] in its *Compendium of Gender Studies in Africa* [28].

The WHC is grounded in empowerment and feminist theory to emphasize the inverse relationship between substance abuse and personal power and that reduced personal power leads to increased risk behaviors and victimization. Two intervention sessions are held approximately 1 week apart, with each session lasting about 1 h. Sessions can be conducted in groups, workshops, or one-on-one individual sessions. Key elements of the sessions educate participants about the risks of AOD use and how AOD use and sexual risk relate to HIV for women. The WHC also teaches risk-reduction methods such as proper condom use, sexual negotiation, and violence prevention strategies. Women also role-play and rehearse how to use male and female condoms correctly, as well as condom negotiation. At the end of the second session, participants create goals to reduce personal risk. Intervention staff also provide referrals to services and linkages to care.

Following numerous trials in which the WHC demonstrated efficacy, the program was packaged to facilitate widespread dissemination and implementation. The intervention package includes a brief marketing overview for decision-makers and materials for interventionists. It also includes a complete set of intervention materials, the curriculum, and an outreach manual with instructions related to reaching hard-to-reach vulnerable women. Based on the principles of translation frameworks, such as the RE-AIM model [29], the next logical step would be to evaluate the implementation of the packaged WHC in real-world clinical care settings. In this implementation science project, the WHC intervention will be implemented by clinic staff in healthcare clinics and substance use rehab centers as part of standard of care among women living with HIV who use substances. The WHC will address women’s multiple risk-taking behaviors and promote adherence to ART.

### Implementation of the WHC

Prior research suggests the need to adapt and modify evidence-based programs to make them more flexible and amenable to scale-up in real-world settings [13]. The adaptation and modification steps need to be monitored and evaluated to ensure that they support continued fidelity to the overall model and lead to outcomes that are similar, but not necessarily identical, to efficacy trials. A framework for wider scale-up that leads to adoption of an innovation in real-world settings must consider multiple, nested levels—such as patient, provider, setting, organization, and environment—at which implementation and evaluation occur [30]. The approach posits that successful implementation begins with an evidence-based practice, in this case the WHC, to which there is initial training of clinical staff followed by preliminary and setting-specific modifications, then adoption and evaluation of multilevel outcomes. The process begins with formative work to identify any potential barriers to adoption and use of the WHC. Modification, implementation, and evaluation are iterative and interactive processes that form a feedback loop for monitoring service delivery, intervention refinements, implementation strategies, evaluation protocols, and quality of care in a staged progression prior to full implementation. As part of this project, these multiple steps will be implemented to refine and test the WHC program for wide-scale use in healthcare clinics and substance use rehab centers.

## Methods

### Aims and objectives

The overarching goal of this project is to assess the acceptability and feasibility of implementing the WHC in usual care settings and to assess the effectiveness of the intervention as it is implemented. The project has three specific aims. Aim 1 is to undertake qualitative data collection to develop, implement, and assess the appropriateness of a marketing plan or a recruitment strategy to facilitate entry into HTC/healthcare clinics and substance use rehab centers for the implementation of the WHC. Aim 2 is to test and evaluate a modified WHC program that has inputs from the first phase; evaluation will use a stepped-wedge design across the health clinics that provide HTC, ART, TB treatment, and antenatal care matched with substance use rehab programs to determine implementation outcomes (appropriateness, acceptability, adoption, cost, fidelity, feasibility, and sustainability) and service outcomes (comprehensiveness of services and timely service linkages). Aim 3 will assess the impact of the WHC program on patient outcomes at 6-month follow-up.

### Setting

The WHC is being rolled-out sequentially in four HTC/healthcare clinics and four outpatient substance use rehab centers located in selected disadvantaged communities (townships) in Cape Town. These sites were identified and approved by the City of Cape Town Health Department. Each HTC/healthcare clinic was paired with a substance use rehab center based on geographic proximity, and each pair was randomized by computer into four succeeding 6-month implementation cycles where implementation of the WHC is taking place simultaneously at the paired sites.

### Study design

**Fig. 1:**
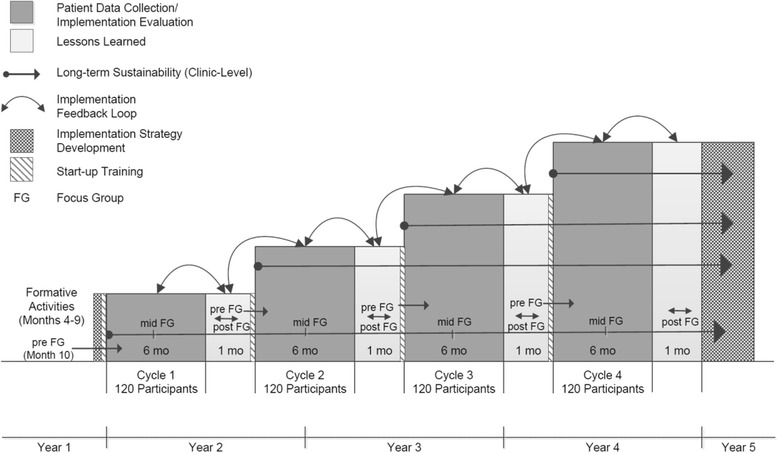
Implementation science study design for the Women’s Health CoOp program

The overall study uses a stepped-wedge design, as shown in Fig. 1. This design lends itself to implementation science research adaptation and mixed methods [31]. It also has several logistical and scientific advantages, as it involves incremental execution of the intervention; allows continued measurement of key process and outcome indicators; accommodates periods of feedback loops; allows for retrospective evaluation of sustainability; and accounts for temporal trends, such as changes in ART regimens (i.e., universal coverage) or funding for healthcare that may impact implementation. Our iterative approach will incorporate lessons learned at each step, making the integration process more efficient and conducive to identifying aspects of the clinic environment and the WHC that are acceptable, scalable, and sustainable.

As depicted in Fig. 1, this study has 4 cycles spanning several years. Each implementation cycle of the WHC program lasts 6 months. Based on the findings from each cycle, we undertake a brief formative “lessons learned” evaluation. These periods between cycles inform backward- and forward-implementation strategies through mixed methods of brief staff surveys and focus group discussions (FGDs), allowing for modification of the WHC while preparing for training in the next cycle. Pre-, mid-, and end-of-implementation FGDs are conducted to inform the modifications, including training materials, curriculum, and implementation data collection. Pre-implementation FGDs involve setting the stage for entry into the clinics, so that barriers and challenges can be addressed. Mid-implementation FGDs explore initial results and help determine if additional modifications to the intervention are needed. End-of-implementation FGDs with clinic staff and patients explore satisfaction with the intervention and concerns with the program’s sustainability. This format provides back-and-forth feedback and dialogue with clinic staff on the implementation processes and outcomes for further refinement. Consequently, the clinics randomized to Cycle 1 will have the longest post-intervention observation period, with implementation sustainability measured through fidelity checks and quantitative surveys. Conversely, based on lessons learned throughout the earlier cycles, sites in Cycle 4 benefit from an intervention that is more refined and in the most scalable form.

### Study procedures

#### Data collection: formative phase

The formative phase was completed in November 2014. During this phase, we engaged our Community Collaborative Board (CCB)—a group of key stakeholders and policymakers that have been key collaborators on the CoOp studies in South Africa since 2003, staff from the local department of health, and women who use substances from the target communities—to develop a marketing plan and engagement strategy that addressed the potential challenges and barriers to integrating the WHC into existing HTC/healthcare clinics and substance use rehab centers in Cape Town. These informants helped identify potential challenges to integrating the WHC into usual care settings. Methods of engagement included expert interviews with regional and local policymakers and FGDs with staff from HTC/healthcare and substance use rehab centers. Information from the formative phase led to important adaptations to the WHC model. In particular, we were able to identify the need to implement extensive recruitment strategies in the community because many of the women who were living with HIV and using substances were not currently visiting health facilities [32].

#### Data collection: implementation phase

We are using a Type 2 hybrid trial design that emphasizes both clinical effectiveness and implementation outcomes, with the eventual goal of more rapid translation and uptake to usual care settings [33]. Consequently, data collection will include repeated measures at the clinic and patient levels.

### Clinic-level data collection

Prior to integrating the modified WHC program into the facilities, clinic staff are identified and presented with information on the WHC intervention. They then complete a survey to assess organizational readiness for change, including perceived barriers to change, acceptability, feasibility, and appropriateness of implementing the WHC in these facilities. This survey is then re-administered at the end of the implementation cycle. This process will be repeated at subsequent cycles. To assess long-term sustainability of the WHC, we will complete a brief checklist 6 months after the end of each cycle. These post-intervention sustainability checks will be repeated after each cycle such that Cycle 1 facilities have the longest post-intervention observation period on sustainability.

### Patient-level process and data collection

The patient-level study includes a baseline visit and a 6-month follow-up visit to evaluate intervention effectiveness. Eligibility criteria include (1) being between the ages of 18 and 45 years, (2) self-reporting use of at least one drug, including alcohol, at least weekly during the previous 3 months, (3) reporting unprotected sex with a male partner in the past 6 months, (4) having a positive verifiable HIV test result, (5) reporting the intention to remain in the area for at least the next 6 months, (6) providing contact information, and (7) being willing to participate in alcohol and other drug screening.

A field team hired by Kheth’Impilo, the local nongovernmental implementing partner, leads the outreach and data collection activities. Trained outreach workers operate as recruiters of women within clinics and surrounding communities and track eligible women for the study, while data capturers collect patient-level data from participants at the baseline and 6-month follow-up appointments. These are well-established methods from other studies conducted in the region [34, 35]. Once participants are enrolled, clinic staff trained and certified in the WHC offer the adapted intervention to all women enrolled and to other women in their clinics who may be interested in participating, so as to operate as a real-world setting.

At each data collection visit, a member of the research/implementing team administers a face-to-face interview using a secure tablet with skip patterns and quality-control checks programmed into the interview. The study instrument includes measures of socioeconomic status, sex risk behaviors, AOD use, substance abuse treatment readiness, ART use and barriers to use, and other self-reported clinical outcomes such as TB and symptoms for sexually transmitted infections. Biological data are obtained from a urine specimen tested for pregnancy and metabolites for recent drug use. Participants also undergo a breathalyzer test for recent alcohol use. The same measures are collected at 6-month follow-up.

HIV testing is not part of the research because it is performed by clinic staff as part of standard clinic operations. As proof of HIV-positive status, participants must provide a clinic-issued document such as an antiretroviral (ARV) card or ARV medication with identifiable data linking to the participant, or they must consent the research staff to access their hospital records for their results. Viral load is also collected at baseline and 6-month follow-up from the South African National Health Laboratory Service (NHLS) database in the clinics. Viral load testing is also part of the standard of care and the clinics routinely perform HIV viral load testing 6 months after a patient begins taking ART and then 12 months later; however, these intervals may vary by clinic. Viral load test information will be used to validate self-reported ART adherence.

In each cycle, the objective is to enroll 120 women (60 from a HTC/healthcare clinic and 60 from a substance use rehab center) for a sample total of 480, and to follow each enrolled participant at 6 months, with an expected follow-up rate of 90%. At the end of the 6-month follow-up period, we will assess effectiveness of the program using pre-post comparisons to determine whether women reduced their AOD use, are adhering to their ARV regimen, have reduced their sex risk behaviors (e.g., using a condom at every sex act; less transactional sex), and have experienced less to GBV. At the end of each cycle, we will conduct FGDs with a subsample of women who participated in both workshops of the intervention and completed their 6-month follow-up appointment. These study activities will enhance our understanding of women’s satisfaction with the WHC program and suggest modifications to the program for broader acceptability and implementation that will be incorporated into the next cycle.

### Data management and quality assurance

To protect confidentiality, the study assigns each participant a unique study identification code (ID). This ID is the only link between the behavioral and biological data and the identifying information collected for locating participants for their follow-up interviews. Locator information is stored separately from other data in a double-locked file cabinet in a locked room with restricted access. Data collection for this study is conducted by highly trained, local implementing staff that develop a rapport with the study participants to engender trust and elicit the most accurate data possible. Data are transmitted each day from the field site to secure servers in the United States. The US-based data manager reviews additional automated quality control checks that the software generates each day. If any critical inconsistencies are noted, the data manager contacts the field project manager who works with the field staff members to resolve the inconsistencies. The principal investigator, other members of the research team, and the field staff also receive daily activity reports.

### Adverse events and severe adverse events

Adverse events (AEs) or severe adverse events (SAEs) related to data collection or any other research activities are reported to the principal investigator and co-investigators within 24 h, and the funding agency and the Institutional Review Board within 48 h, with appropriate action taken immediately. HTC/healthcare clinics and substance use rehab centers follow their standard procedures, including distressed respondent procedures, for any AEs related to the participants that they treat. The study does not interfere with the clinics’ standard operating procedures.

### Evaluation of outcomes

#### Clinic operations and implementation indicators

Prior to the initiation of each cycle, the master trainer from Kheth’Impilo trains the clinic staff on implementation of the WHC program for 2 weeks prior to implementation launch. Clinic staff involved with the WHC receive intensive, manual-supported training on the WHC and must role-play and rehearse the protocol. Following training, the trainer provides ongoing monitoring, coaching, and mentorship for the 6-month follow-up period. The implementing research staff receive training on data collection and methods; clinical manifestations of drug abuse and HIV; issues surrounding confidentiality, especially within the communities where there is concern about local gossip as reported from the formative activities; to ensure that participants are not intoxicated; and to address any HIV-related concerns during research activities [32].

During each cycle and between cycles, we will quantitatively and qualitatively assess barriers and facilitators to the WHC implementation at the clinic level from the perspectives of interventionists, administrators, and other healthcare providers involved in the WHC program. Modifications identified through the cyclical assessments are incorporated into the training of trainers' materials to support continued adaptation and refinements for long-term sustainability. To assess costs, clinic staff interventionists will record the number of minutes spent on various WHC-related tasks, such as preparation, conducting intervention sessions, and referrals.

To measure the implementation processes and outcomes, we will undertake quantitative data collection with providers and review implementation records to assess fidelity of program implementation. Fidelity checks on a random basis will be conducted on the actual groups conducted. Clinic interventionists and other clinic staff who interact with patients participate in the pre-, mid-, and post-implementation FGDs and also complete the self-administered pre- and post-clinic surveys. We will assess seven implementation outcomes to determine program success, as proposed by Proctor and colleagues [13]. Not all implementation outcomes will be assessed across each of the four cycles; however, the protocol for measuring implementation outcomes will remain constant at each cycle. Some of the intervention outcomes will be measured quantitatively and others will be measured qualitatively. The standard implementation outcomes and their measurement methods are outlined in Table 1.

**Table 1:** Standard implementation outcomes and their measurement methods

Implementation outcome	Description	Measurement	Data source	Time point
Appropriateness	The extent to which the WHC^a^ is congruent with the culture of the clinic setting	Focus group discussions	Clinic staff, community women, administrators	Formative
	Organizational Readiness	Texas Christian University organizational readiness for change [46]	Interventionists and administrators	Pre-implementation
	Comprehensive Services Linkage Referrals	Self-report	Participants of the WHC	6-month follow-up
	Comprehensive Services Linkage Referrals	Research staff report	Clinic and research staff	Daily
Acceptability	Feedback on the modified WHC	Focus group discussions	Clinic staff and administrators	Multiple time points from mid- to post-implementation
	Client satisfaction	The Client Satisfaction Questionnaire (CSQ-8) [47]	Participants of the WHC	6-month follow-up
Adoption	Current state of implementation of the WHC and challenges to implementing the WHC	Focus group discussions	Clinic staff	Mid- and post-implementation
Cost	Costs associated with implementing the WHC	Clinic-Patient Contact form [48]	Clinic staff	Throughout the implementation cycle
Feasibility	Exposure to and retention of the WHC	Number of WHC modules being delivered	Clinic staff	Throughout the implementation cycle
	Coordination of services related to WHC (e.g., ART)	Percentage of women who are referred to and seek out related services	Participants of the WHC	6-month follow-up
Fidelity	The extent to which interventionists are conducting the WHC as intended	Observer rating forms	Research staff	Weekly
Sustainability	The extent to which the WHC is being implemented as a standard of practice	Self-administered survey	Clinic staff	Every 6 months after exit from clinic until end of study

^a^WHC = Women’s Health CoOp

### Modifications to protocol

The team has made modifications to the protocol to strengthen the study design and facilitate implementation. Table 2 summarizes these changes, which were all approved by the appropriate ethical boards.

**Table 2:** Modifications to the protocol and intervention adjustments/considerations

Date	Amendment
August 2015	Required a digital photo-capturing method to document drug and pregnancy tests, to be later verified by the project manager
November 2015	Adopted the use of clinic-issued ARV cards and ARV medication as proof of participant’s HIV-positive status in the absence of patient records
November 2015	Allowed the use of outreach assistants (who are different from the outreach field staff) to identify locations to reach potential participants.
August 2016	Added collecting HIV viral load tests results from participant clinics to validate self-reported ART adherence
August 2016	Added a sustainability questionnaire to be administered to clinic staff, to monitor sustainability of the WHC Intervention Adjustments/Considerations: The drugs of abuse needed some adjustments for the local context from other regions; personalized plan used in previous studies was not found to be feasible because of concerns about time limitations; there was not time for case-management or interventionist follow-up from the sessions; transportation and childcare would have to be considered; training clerks and community healthcare workers such as nurses/clinicians were too busy; fidelity checks cannot always be audio-recorded because of steady intercom usage.

### Power

We used the Stata command, *steppedwedge* [36], which is based on the equation developed by Hussey and Hughes [37] to calculate the detectable difference between participants’ adherence before and after their exposure to the intervention, given a sample size that is feasible and realistic in these clinic settings (*n* = 480; or 120 participants per cluster). We conducted two analyses based on the following parameters: 120 participants per cluster, 240 observations per cluster, and power of .80. The first analysis was used to calculate the detectable difference between participants’ self-reported percentage of ART adherence during the past month before and after the intervention. No previous research has estimated the percentage of ART adherence among South African women who use substances since the government approved the provision of ART to all people living with HIV, regardless of CD4 count. Consequently, we based our analyses on a national estimate of the average percentage of ART adherence in South Africa since the ART rollout (87%; SD = 17.28). Based on this estimate, we will be able to detect an increase or decrease in adherence of 4% or greater. Next, we calculated the detectable difference between the proportion of adherent (i.e., report 95% or greater adherence or are virally suppressed) participants before and after their exposure to the intervention. We based this calculation on previous research that has reported on the proportion of people who engage in substance use that are adherent to ART (56%) [38] and national estimates indicating that 55% of all people living with HIV are virally suppressed [39]. In regards to self-report adherence, we estimate that we will be able to detect a difference of 13% or greater. Similarly, we also estimate that we will be able to detect a difference of 13% or greater to examine the change in the proportion of women who are virally suppressed before and after the intervention.

### Analysis

The analysis will treat count outcomes as Poisson distributed. A number of multilevel factors can impact the outcomes, including site/clinics, city, time, and the type of the site (HTC vs. substance use rehab). Because individuals are clustered within sites and sites are clustered within a city, we need to consider the variance inflation factor that results in this clustering. We considered a number of approaches: using random effects, fixed effects, or Generalized Estimating Equations (GEE) considering population-averaged methods. Although a random effects model for sites seems a natural approach, we needed to consider fixed effects of the city and of the site type, which would leave little power left to estimate the random effects distribution. Consequently, we will also use two other methods, GEE and the fixed effects model, to assess sensitivity by comparing the results across these models.

We will use Stata Release 14 (College Station, TX) and SAS version 9.2 (Cary, NC). Data will be examined to determine the extent to which they meet appropriate distributional assumptions. We will use methods described by Ecob and Der to identify outliers in longitudinal models and use methods of case downweighting when extreme cases are identified [40]. Missing data will be handled using multiple imputation methods [41]. The patient-level analyses will be organized into three groups to examine (a) successful reduction of risk behaviors targeted by the WHC (i.e., effectiveness), (b) patient satisfaction, and (c) increasing the number of women receiving ART and adhering to ART, and being admitted to substance use rehab.

To assess effectiveness, the analysis will compare changes from baseline to 6-month follow-up on the amount of alcohol consumption, biological and self-reported drug use, condom use at last sex, number of sex partners over the past 6 months, and values from scales that measure relationship power and communication. We will start with descriptive analyses, including measures of central tendency, and dispersion (e.g., means, medians, proportions, standard deviations). Bivariate analysis will include paired t-tests, Wilcoxon rank-sum, and tabular statistics and the chi-square test. For multivariable analysis, the xtlogit and xtgee function will be used to specify random effects or fixed effects, and will assume that the correlation matrix is exchangeable. The models will control for other covariates identified in bivariate analyses or hypothesized a priori (e.g., condom use is correlated with substance use). One of the most important covariates in the model will be measures of patient satisfaction. Model performance and fit will be assessed with incremental elimination of variables, likelihood ratio tests, and changes in the Akaike information criterion (AIC) and Bayesian information criterion (BIC) values.

Patient satisfaction analyses will be predominately descriptive to determine if satisfaction improves with each cycle, varies by type of clinic, and by participant characteristics (e.g., alcohol use, pregnancy status).

We assume that the prevalence of patients receiving or adhering to ART or receiving treatment for substance abuse are Poisson distributed and increase from baseline to follow-up. We will use a similar approach outlined in the effectiveness analysis for bivariate and multivariable analyses. Variables considered for multivariable analysis will include patient characteristics, measures of patient success with the WHC, patient satisfaction, and clinic-level characteristics (e.g., type of clinic, provider satisfaction).

## Discussion

Multiple EBIs exist to help reduce HIV risks in low- and middle-income countries. However, only about 14% of EBIs are ever incorporated into standard practice. For those 14%, it takes an average of 17 years from the time they are developed and tested to the time they are implemented widely [42]. Successful translation of EBIs into practice must be informed through implementation science research, which seeks to document the process of uptake, translation, and implementation of these EBIs into real-world settings.

This article describes the study protocol for a project to assess the feasibility and acceptability of implementing the WHC in HTC/healthcare clinics and substance use rehab centers. This project uses a stepped-wedge design that permits continued adaptation and modification of the process to ensure that lessons learned in each cycle are incorporated to enhance the sustainability and scalability of the intervention for broader implementation.

Undertaking these types of projects for implementing EBIs into new settings is an important goal of future HIV programs because the evidence on how to implement efficacious interventions effectively remains scarce [43]. Implementation research projects, such as this, may be particularly critical in helping UNAIDS reach its 90–90-90 goal for HIV testing, treatment, and viral suppression. Additionally, projects like this can provide guidance for how to scale up efficacious programs that identify women who may be unaware they are HIV positive, link them to testing and treatment, and support them to adhere to ART.

Numerous prior interventions that demonstrated efficacy in randomized trials are not scalable or sustainable. Consequently, undertaking studies to adapt and test variations of these interventions are needed to determine the best strategies for implementing them in community care settings within disadvantaged communities in South Africa. However, as noted, real-world settings present practical problems and most care providers need to be convinced that these interventions will improve care without increasing the burden on the healthcare team. Consequently, intervention developers should assess the strengths and limitations of their anticipated implementation setting when developing and attempting to implement and scale-up interventions.

One particularly important aspect of this project is the marketing campaign to achieve buy-in from providers and patients prior to implementing the intervention. Clinical staff recognize that to reach the 90–90-90 goal more women need to get tested, as many are unaware of their serostatus and there are many new incident cases among women of childbearing age [17]. However, one thing that some care providers and staff may not have considered is that substance use in South Africa is a serious problem, especially alcohol use during pregnancy [44, 45]. These interacting forces, along with high prevalence of GBV within relationships and communities, necessitate a gendered intervention for women, especially women living with HIV. Our WHC marketing materials highlight that HIV, AOD, and GBV are critical problems for women and introduces the WHC intervention as a program that addresses these issues to promote adherence to ART. Clinic staff want good clinical outcomes and such an intervention could be a win-win for all if implemented successfully, especially if women felt comfortable discussing these issues.

South Africa has a long history of class, ethnic, and gender inequality. Many of these disparities are still prominent in the communities where these women live, and this project presents an opportunity to help empower women through an intervention offered in a usual care setting if successfully sustained.

### Trial status

The study is now in the field, with further modifications necessary. The first modification involved identifying women who use substances and are living with HIV, because many women are unwilling to admit that they are living with HIV or that they are using substances. Additionally, they may be unwilling to be tested because they have been treated poorly and are afraid to go to health services. Consequently, to increase access to healthcare services for study participants, the research team had to provide training to clinic staff and outreach workers to educate them about stigma surrounding HIV and substance use. Further, the electronic database systems to verify HIV status for those who had already tested were out of date (e.g., some names and dates of birth were not accurate based on the information given by the participants). Consequently, the research team established alternative protocols to verify HIV status.

## Abbreviations

AE: Adverse event
AIC: Akaike information criterion
AOD: Alcohol and other drugs
ART: Antiretroviral therapy
ARV: Antiretroviral
BIC: Bayesian information criterion
CCB: Community Collaborative Board
CSQ: Client Satisfaction Questionnaire
EBIs: Evidence-based interventions
FGD: Focus group discussion
GBV: Gender-based violence
GEE: Generalized Estimating Equations
HTC: HIV testing and counseling
NHLS: National Health Laboratory Service (South Africa)
SAE: Severe adverse event
UNAIDS: The Joint United Nations Programme on HIV/AIDS
USAID: United States Agency for International Development
WHC: Women’s Health CoOp
